# Supplementary material for: Anti-Gr-1 Antibody Provides Short-Term Depletion of MDSC in Lymphodepleted Mice with Active-Specific Melanoma Therapy
Source: Vaccines (Basel). 2022 Apr 4;10(4):560. doi: 10.3390/vaccines10040560 (PMC9032646; doi:10.3390/vaccines10040560)
Supplement: Supplementary file 1 [file vaccines-10-00560-s001.zip › Suppl Mat.pdf]

Supplementary figures:

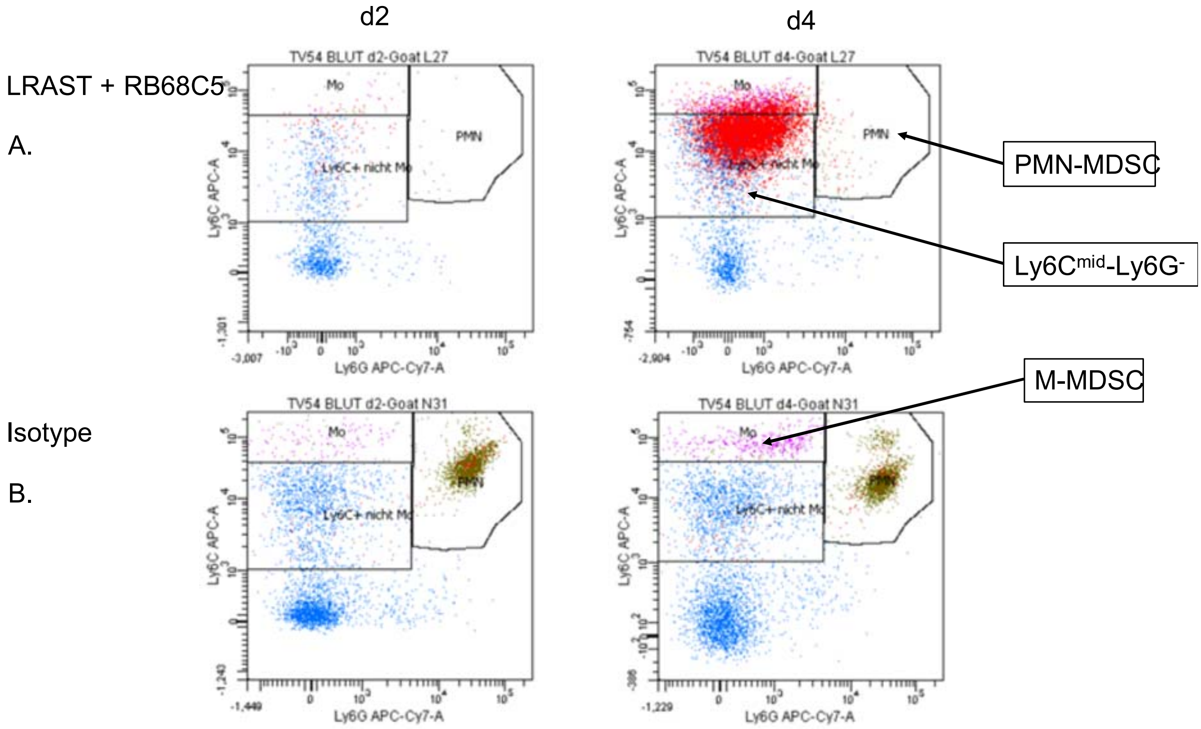


**Figure S1: Recurrence of PMN-MDSC.** Representative dot plots showing PMN-MDSC, M- MDSC and Ly6G$^{-}$ Ly6C$^{mid}$ cells according to Ly6G/Ly6C-staining of CD45.2$^{+}$ CD11b$^{+}$ MHCII$^{-}$ cells. Cells were analyzed from blood of D5-melanoma carrying mice with LRAST + RB6-8C5 treatment (a), or no treatment (isotype alone, control, b) on day 2 and day 4 after treatment initiation. Red dots represent RB6-8C5-bound cells, as indicated by secondary antibody staining (PE-conjugated goat anti-rat IgG).

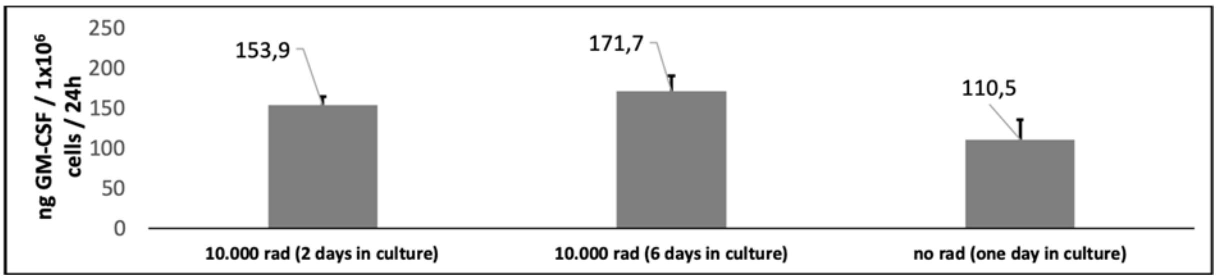


**Figure S2: In vitro GM-CSF production of D5G6 cells.** We confirmed durable GM-CSF secretion of D5G6 cells after irradiation with 10.000 rad compared to non-irradiated D5G6 cells. Cells were kept in culture for a maximum of 6 days. GM-CSF production was evaluated with GM-CSF-ELISA and normalized to 1x10$^{6}$ cells and 24 hours.

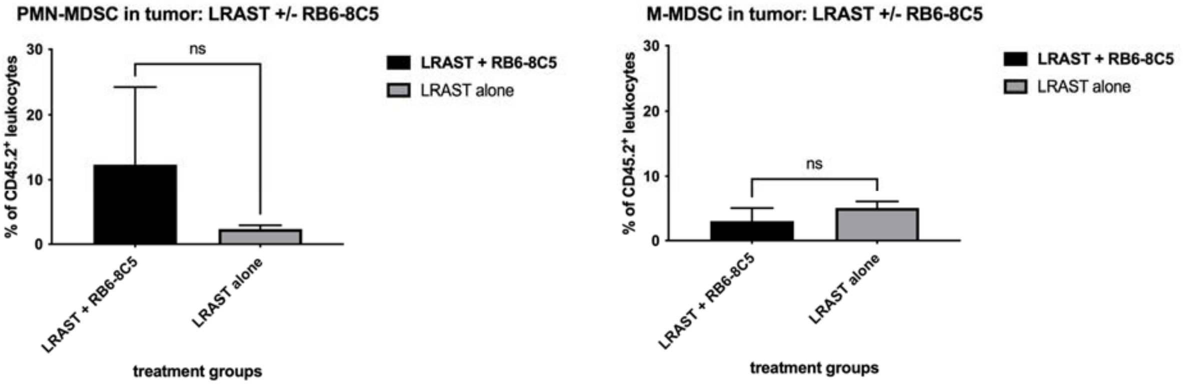


**Figure S3: Tumor-infiltrating PMN- and M-MDSC after LRAST +/- RB6-8C5.** Flow cytometric analysis of D5-tumor tissue reveals PMN- (left) and M-MDSC (right) as % of CD45.2$^+$ leukocytes from mice treated with LRAST + RB6-8C5 ($n$ = 4) or LRAST alone ($n$ = 3). Mice were euthanized and tumors harvested for FACS-analysis (LRAST + RB6-8C5: 26/29/35 and 37 days after treatment initiation; LRAST alone: 29/32 days after treatment initiation). Bars indicate means with SD. For statistics Student´s t-test was performed.
